# Towards a machine-learning assisted non-invasive classification of dengue severity using wearable PPG data: a prospective clinical study

**DOI:** 10.1016/j.ebiom.2024.105164

**Published:** 2024-05-29

**Authors:** Stefan Karolcik, Vasileos Manginas, Ho Quang Chanh, John Daniels, Nguyen Thi Giang, Vu Ngo Thanh Huyen, Minh Tu Van Hoang, Khanh Phan Nguyen Quoc, Bernard Hernandez, Damien K. Ming, Hao Nguyen Van, Tu Qui Phan, Huynh Trung Trieu, Tai Luong Thi Hue, Alison H. Holmes, Louise Thwaites, Tho Phan Vinh, Sophie Yacoub, Pantelis Georgiou, Vietnam ICU Translational Applications Laboratory (VITAL) Investigators

**Affiliations:** a Centre for Bio-Inspired Technology, Imperial College London, South Kensington Campus, London, SW7 2AZ, United Kingdom; b Centre for Antimicrobial Optimisation, Imperial College London, Hammersmith Campus, London, W12 0NN, United Kingdom; c Oxford University Clinical Research Unit (OUCRU), Hospital for Tropical Diseases, Ho Chi Minh City, 700000, Viet Nam

**Keywords:** Dengue, Photoplethysmography (PPG), Deep learning

## Abstract

**Background:**

Dengue epidemics impose considerable strain on healthcare resources. Real-time continuous and non-invasive monitoring of patients admitted to the hospital could lead to improved care and outcomes. We evaluated the performance of a commercially available wearable (SmartCare) utilising photoplethysmography (PPG) to stratify clinical risk for a cohort of hospitalised patients with dengue in Vietnam.

**Methods:**

We performed a prospective observational study for adult and paediatric patients with a clinical diagnosis of dengue at the Hospital for Tropical Disease, Ho Chi Minh City, Vietnam. Patients underwent PPG monitoring early during admission alongside standard clinical care. PPG waveforms were analysed using machine learning models. Adult patients were classified between 3 severity classes: i) uncomplicated (ward-based), ii) moderate-severe (emergency department-based), and iii) severe (ICU-based). Data from paediatric patients were split into 2 classes: i) severe (during ICU stay) and ii) follow-up (14–21 days after the illness onset). Model performances were evaluated using standard classification metrics and 5-fold stratified cross-validation.

**Findings:**

We included PPG and clinical data from 132 adults and 15 paediatric patients with a median age of 28 (IQR, 21–35) and 12 (IQR, 9–13) years respectively. 1781 h of PPG data were available for analysis. The best performing convolutional neural network models (CNN) achieved a precision of 0.785 and recall of 0.771 in classifying adult patients according to severity class and a precision of 0.891 and recall of 0.891 in classifying between disease and post-disease state in paediatric patients.

**Interpretation:**

We demonstrate that the use of a low-cost wearable provided clinically actionable data to differentiate between patients with dengue of varying severity. Continuous monitoring and connectivity to early warning systems could significantly benefit clinical care in dengue, particularly within an endemic setting. Work is currently underway to implement these models for dynamic risk predictions and assist in individualised patient care.

**Funding:**

EPSRC Centre for Doctoral Training in High-Performance Embedded and Distributed Systems (HiPEDS) (Grant: EP/L016796/1) and the 10.13039/100010269Wellcome Trust (Grants: 215010/Z/18/Z and 215688/Z/19/Z).

**Research in context**

**Evidence before this study**

We searched Google Scholar for published articles available in English using the keyword groups (“deep learning” or “machine learning” or “artificial intelligence”) and (“photoplethysmography” or “PPG”) and (“dengue” or “hemorrhagic fever”) up to May 1st, 2023. While the field of applying machine learning to interpret PPG waveforms has been on the rise, there is only a limited amount of publications exploring the specific relationship between dengue and PPG. The identified publications focused on extracting dengue surrogates like haemoglobin concentration from the PPG data and did not fully explore the link with dengue.

**Added value of this study**

In this study we leverage photoplethysmography, a cost-effective and non-invasive optical monitoring technology, to perform a large-scale investigation into the monitoring of dengue fever and its subsequent severity classification. With more than 1700 h of continuous waveform data from over 150 patients with dengue, we have compiled a dataset to tackle the burden of this disease with cost-effective solutions. Through a series of experiments, we have developed a set of machine learning models capable of accurate classification of dengue severity states using segments of PPG waveform alone.

**Implications of all the available evidence**

The achieved performance of the models indicates exceptional accuracy in selecting between ill states and has the potential to significantly improve the current standard of care for this difficult-to-manage disease. Its large, hospital-acquired dataset puts it in a unique position to deliver a modern and cost-effective patient management tool to LMIC countries suffering from endemic dengue infections. Accurate severity state classification has the potential to inform attending clinicians of the effectiveness of delivered care and identify at-risk patients in already overcrowded health systems.

## Introduction

Dengue has emerged in the last two decades as the most common vector-borne viral infection globally. It has been estimated that 96 million clinically apparent dengue infections occur worldwide each year, as well as a further 300 million asymptomatic infections.^1^ This disease imposes a considerable strain on healthcare resources in endemic countries. Dengue causes a wide spectrum of clinical syndromes with the majority of patients experiencing a mild self-limiting febrile illness which does not require hospital admission. A minority, however, develop severe dengue, which is characterized by vascular leak, and hypovolaemic shock and requires urgent medical treatment.^2^ No effective antiviral agents or definitive therapeutics are available to treat dengue, and current management strategies rely on close monitoring of disease progression in order to provide prompt supportive treatment.^3^

Predicting the individual patients who go on to develop severe dengue is challenging. Clinical evaluation of patients with dengue in early illness can help identify signs which are associated with severe disease.^3^ The patients who are at increased risk of severe dengue are commonly hospitalized in order to undergo frequent clinical assessments in anticipation of the development of shock, bleeding and/or organ impairment.^3^ Evaluation of vital signs parameters including heart rate, blood pressure, pulse pressure and blood hematocrit is crucial in dengue monitoring since they provide information regarding volume distribution and hemoconcentration.^2^ However, the processes of clinical monitoring are inherently demanding on healthcare resources in terms of time and staffing. During the peak season of dengue, high-quality medical equipment required to collect these parameters can often be in short supply within low-income and middle-income (LMIC) settings, limiting the timeliness and effectiveness of healthcare interventions. Alternative approaches which can closely monitor patients and give timely warnings of physiological deterioration in dengue are needed.

One approach to providing robust physiological monitoring for patients in LMICs is through the use of low-cost wearable sensors. The non-invasive nature, low cost, and ability for continuous real-time monitoring and connectivity of wearables can lead to improved care and outcomes of patients admitted to the hospital.^4^ Such wearables commonly employ photoplethysmography (PPG), a well-established technique for obtaining information about blood volume changes during the cardiac cycle and monitoring heart rate by measuring light absorption increases associated with the systolic increase in arterial blood volume.^5^ Recently, machine learning algorithms have been developed to measure fluid volume status using a parameter called the compensatory reserve index (CRI) before any signs of hypotension, using PPG alone in patients with haemorrhagic shock,^6^ and dengue.^7^ As PPG is commonly employed by wearable devices, this approach could provide robust physiological monitoring for patients with dengue.

Deep learning convolutional neural networks have been the model of choice when analysing raw PPG datasets,^8^^,^^9^ however, only limited work was done to explore the specific relationship between infectious diseases and waveform features.^10^ To our knowledge, studies focusing on dengue management generally use traditional methods such as decision trees,^11^ multiple linear regression^12^^,^^13^ and gradient boosting methods.^14^ These methods are usually applied to manually collected clinical datasets with low granularity. This opens opportunities to use continuous PPG data to explore the link between waveform features and dengue severity and build paths for approaches to patient management using low-cost, wearable devices.

The study starts by introducing a continuous PPG dataset collected from patients with dengue at the Hospital for Tropical Diseases in Ho Chi Minh City, Vietnam. The dataset was acquired using a commercial wearable finger PPG sensor together with timestamped clinical spot measurements obtained during routine care. Following the dataset collection, we have developed an exploratory machine-learning pipeline to investigate two classification objectives using a set of models with increasing complexity. The primary objective of the study focused on showing that continuous, wearable PPG data acquired from adult patients with dengue contain enough information to determine the severity level while the secondary objective focused on distinguishing between ill and recovered paediatric patients with dengue. Both of these objectives pave the way for cost-efficient patient monitoring approaches for seasonal infections, specifically prevalent in LMIC.

## Methods

### Study design and participants

This observational clinical study was conducted at the Hospital for Tropical Diseases in Ho Chi Minh City, Vietnam and contained 132 adults and 56 paediatric patients at various stages of the disease. Clinical recruitment was performed between June 2020 and April 2022. The sample size for this study was determined by the constraints of a typical clinical practice, reflecting the number of patients diagnosed with dengue available for inclusion.

Continuous waveforms have been collected using a wearable PPG sensor developed by Smartcare Analytics (Oxford, United Kingdom), capable of pulse oximetry with wavelengths at red and IR frequencies. The aim was to capture continuous data for the first 24 h after hospital admission as those are the most critical from a dengue disease progression perspective. The total recording time was reduced for a subset of emergency department patients where data acquisition was stopped before transfer. All raw waveforms were sampled at 100 Hz and saved using the accompanying tablet app.

Patients were recruited within 48 h of admission to the Hospital for Tropical diseases. Adult and paediatric patients who met inclusion criteria were approached and recruited by study staff. Once informed consent and/or assent form was obtained, patients were enrolled to the study. Inclusion criteria required patients with a clinical diagnosis of dengue and aged ≥8 years. Exclusion criteria included: (1) Failure to give informed consent and (2) contraindications to use of monitoring equipment (e.g. prone positioning, allergic to electrodes, could not apply PPG device due to finger/toe disability).

Collected clinical data included clinical examination, vital signs, laboratory tests, and treatment received at enrolment, over the first 24 h after enrolment, and then daily up to 5 days or until hospital discharge, whichever was sooner. Measurements of blood pressure, pulse rate, respiratory rate, body temperature and oxygen saturation were taken as part of standard care. These were recorded from medical notes in the case report forms (CRFs) for all patients enrolled in the study. Information regarding routine patient fluid intake, inotropic therapy, results of blood tests (e.g. haematocrit, platelet count) and changes in clinical management were also captured in the CRFs. For all patients, sex was determined from medical records.

#### Data quality

The raw PPG signal acquired from the wearable is processed through a 2-stage pipeline to determine if it has sufficient quality for further analysis. In the first stage, a 4th order Chebyshev II filter with a 0.15 20 Hz passband is used. This was determined to be an optimal bandpass filter type for raw PPG signal in 15. The second stage uses two signal quality indices ($Z_{SQI}$ and $M_{SQI}$) introduced in 16 to accept or reject each filtered signal segment. The zero-crossing rate, or ($Z_{SQI}$), counts the number of times the signal passes the origin as defined in Equation (1). A number of zero crosses in a filtered clean signal segment is directly proportional to heart rate and therefore a signal is deemed acceptable if $Z_{SQI}$ lies within the 45–120 bpm range.

(1) $$Z_{SQI}=\frac{1}{N}\sum_{n\;=\;1}^{N}I\left\{y<0\right\}$$

Matching of multiple systolic wave detection algorithms, or $M_{SQI}$, calculates the agreement between two different peak detector algorithms following the definition in.^16^ The Equation (2) illustrates this approach, where $S_{PD1}$, $S_{PD2}$ represent the set of peaks from the adaptive threshold method and Billauer's method respectively. The acceptable segment threshold for $M_{SQI}$ was set to 0.9 to allow small disagreements between the algorithms.

(2) $$M_{SQI}=\frac{\left(S_{PD1}\cap S_{PD2}\right)}{S_{PD1}}$$

The final decision if the patient data should be included in the subsequent feature extraction and model training is made based on the overall proportion of bad-quality segments. If for a given patient, the proportion of noisy segments is more than 10%, it is removed from the cohort.

### Machine learning pipeline

**Fig. 1:**
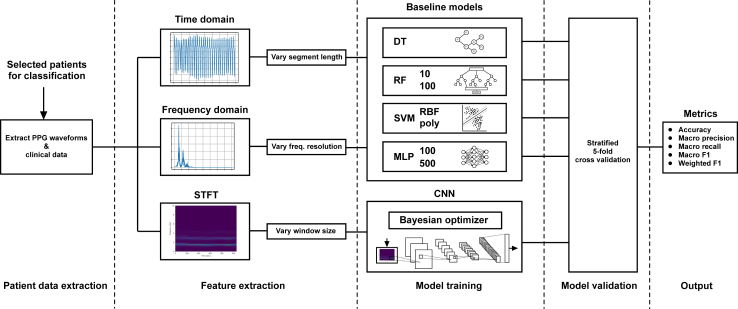
Illustration of the machine learning pipeline used in this work. It starts by extracting relevant patient data (cleaned and filtered) according to the clinical question. The data is then passed through 3 separate feature extraction algorithms feeding their respective models. Variable feature extraction parameters allow further model optimization. In the next stage, the baseline and CNN models are trained. These are done sub-sequentially to evaluate performance improvements. Lastly, each model is separately validated using 5-fold cross-validation before collecting the final performance metrics.

Due to the size of the feature space of the dataset, we have conducted a series of experiments of increasing complexity to develop a suitable model for the classification of disease severity from wearable PPG data as illustrated in Fig. 1. Furthermore, the same approach was used to develop a model for classifying the difference between acute disease and follow-up recordings 3 weeks after the illness onset in paediatric patients. Due to the potentially stressful nature of conventional medical treatments, some intrinsic differences in base modalities like heart rate were expected between these classes, pointing to a likely separability of the dataset. By employing feature extraction pipelines of varying complexities, we were able to investigate the effects of different signal representations on the classification capabilities of the model. The work started by implementing baseline machine learning models to evaluate the performance in the time domain and frequency domain representation of the PPG signal. We have then evaluated deep-learning CNN models using the time-frequency representation of the signal to improve the performance further.

The primary objective of the study was to develop a suitable model for dengue severity classification in adult patients. The secondary research objective was to show that raw PPG data can be used to differentiate between the healthy (post-disease follow-up) and ill (severe dengue) states in paediatric patients.

### Feature extraction

Depending on the complexity of the underlying model, three different FE approaches have been implemented. In the first iteration of baseline models, the PPG waveform is used in its time-domain representation right after the filtering stage. For each of the segments of length n, the feature map will be an array-like structure holding n subsequent signal values. This approach provides a set of baseline performance metrics, highlighting the relationship between continuous PPG signal and the investigated research question.

The frequency-domain signal representation uses half-spectrum FFT. Given the sampling frequency of 100 Hz, the spectrum is defined for [0–50]Hz. However, since the signal is band-pass filtered with a cut-off frequency at 20 Hz, only the magnitude of the spectrum in the range [0–20]Hz is used as illustrated in the Supplemental Figure S1a.

The last FE approach is using time-frequency representations based on the *scipy*^17^ short-time Fourier transform (STFT) implementation. This process splits the signal into n windows, computing the FFT for each and then stacking them into a 2D image to form a feature set. Supplemental Figure S1b illustrates a computed STFT for a segment of PPG signals limited to the [0–10]Hz range for better visualisation.

### Model architecture

#### Baseline models

The baseline models aim to give initial performance metrics of the dataset, allowing comparison-based evaluation of the complex CNN-based models. The collection of baseline models consists of a Decision Tree (DT), Random Forest (RF), Support Vector Machine (SVM), and Multi-Layer Perceptron (MLP) classifiers. Each baseline model has been implemented using the default parameters from the sci-kit learn^18^ Python library to form the first runnable version. We then vary one hyper-parameter per model architecture to evaluate the differences in performance and pave the way for further model optimisation efforts. The full overview of baseline models architecture and hyper-parameters varied is shown in Table 1.

**Table 1:** Collection of baseline models used for experimentation; Total of 8 models derived from 4 model types and through the alteration of one main hyperparameter.

Model Type	Hyperparameter	Value	Abbreviation
Decision Tree	–	–	DT
Random Forest	Number of Estimators	10	RF (10)
Random Forest	Number of Estimators	100	RF (100)
SVM	Kernel Function	RBF	SVM(RBF)
SVM	Kernel Function	Polynomial	SVM(poly)
MLP	Number of Neurons	10	MLP (10)
MLP	Number of Neurons	100	MLP (100)
MLP	Number of Neurons	500	MLP (500)

#### CNN models

We used Bayesian optimization provided by the *comet.ml* platform to traverse the parameter search space. The ranges for these parameters are defined in Table 2. The number of filters of the convolutional layers is based on VGG16,^19^ increasing the number of filters as the network deepens. Our approach is to double the filters of each consecutive layer while maintaining the same number for the last two layers. A max-pooling layer is placed after each convolutional layer to assist with feature extraction within the CNN, as well as to reduce the computational overhead.

**Table 2:** Model search space, as defined by a number of parameters and a corresponding range of values for each, to be explored through the Bayesian optimisation scheme of the *comet.ml* platform.

Parameter name	Range of values	Range type
Number of CLs	[2, 3, 4, 5, 6]	Discrete
Kernel size	[(3, 3), (4, 4), (5, 5)]	Discrete
Number of DLs	[1, 2, 3]	Discrete
Number of nodes per DL	[4, 8, 16, 32]	Discrete
Learning rate	[5e-5, 5e-3]	Continuous

CL and DL stand for convolutional and dense layer respectively.

### Model evaluation

In all of the experiments, a set of five evaluation metrics is computed: accuracy, macro-precision, macro-recall, macro-F1, and weighted-F1. Where macro-averaging refers to the arithmetic mean across classes and weighted-averaging takes into account the proportion of each class label within the dataset. The F1 score is defined in (3) as the harmonic mean of precision (p) and recall (r). The usage of harmonic mean better reflects extreme values and leads to a low overall F1 score if a model has either very poor precision or very poor recall.

(3) $$F_{1}={\left(\frac{r^{-1}\;+\;p^{-1}}{2}\right)}^{-1}=2\;\times \;\frac{\mathrm{pr}}{p\;+\;r}$$

To obtain a more realistic estimate of performance we employ stratified 5-fold cross-validation throughout all the experiments. The Bayesian optimisation algorithm used for CNN parameter selection runs for 20 iterations to maximize the average weighted F1 score over 5 folds of cross-validation.

### Experimental setup

#### Time-domain features

For time-domain analysis using baseline models, we utilize the theorem proposed in 20 stating that the segment length should be “at least 10 times the wavelength of the lowest frequency bound investigated”. In our case, the lowest frequency bound is 0.15 Hz as indicated in the filtering stage. The corresponding wavelength is, therefore, $1/0.15=6.\bar{6}$ seconds, leading to a segment length of ten times that or just over 1 min long. We further investigate the effects on baseline model performance when we halve this optimal segment length.

#### Frequency-domain features

Using the segment length of $66.\bar{6}$ seconds we compute the half-FFT spectrum and extract the range of interest between [0–20]Hz. To explore the effect of varying frequency resolution on model performance, the FFT array is split into n number of bins, computing a single average value for each. For a half-FFT of the signal sampled at 100 Hz, we have a total of 1333 values in the [0–20]Hz range which gives the upper boundary of 1333 bins. We test the performance of all 9 frequency resolutions when using the factor of a power of two and these are [2, 4, 8, 16, 32, 64, 128, 256, 512, 1024].

#### STFT

In the context of the STFT, the suggested signal length of 10 wavelengths refers to the size of the sliding window, and not to the size of the entire STFT. Therefore, since an STFT is obtained by applying the FFT on consecutive signal windows, we use consecutive 66 second long segments. Due to the limited duration of the follow-up healthy recordings, we relax the requirement of the signal length for that question. The result of the process was a calculation of a window size of 9.86 times the lowest frequency wavelength of interest, providing us with 8 STFT samples for the shortest follow-up recording. We further increase the amount of STFT windows by dividing the calculated sizes for the window size by 2, 4, and 8 in order to generate more training samples. These varied approaches are only applicable to the paediatric follow-up vs ICU stay classification problem due to the short follow-up recordings for some patients. The severity classification uses STFT windows for the original segment length of $66.\bar{6}$ seconds.

### Statistics

The statistical analysis in this study was primarily carried out through the use of machine learning techniques. These were chosen over traditional statistical tools due to the nature of the dataset, which is characterized by complex multidimensional and temporal features. In this setting, machine learning approaches were deemed suitable for handling such high-dimensional data and consequently being able to predict dengue severity based on wearable sensor PPG data.

Two sets of inclusion-exclusion criteria were applied. The first set focused on the study enrolment, where the most common exclusion was a contraindication to using devices. The second set focused on data quality, where patients were excluded if more than 10% of their PPG signal was of poor quality, since noise-corrupted PPG readings do not provide meaningful input to the classification model.

### Ethics

The study was approved by the scientific and ethical committee of the Hospital for Tropical Diseases (HTD), Ho Chi Minh City and by the Oxford Tropical Research Ethics Committee (OxTREC) with reference 522-20 on 23rd April 2020. Informed consent was obtained from the patient or their representative. If the patient was a minor (>8 and <18 years of age), assent form was obtained in addition to parental or guardian consent.

### Role of funders

The funders had no roles in study design, data collection, data analysis, data interpretation, or writing of the report.

## Results

### Study population

For the adult cohort of 132 patients, over 1398 h of continuous raw PPG waveform was included in the primary analysis. This cohort was further split into 3 categories according to the severity of the patient during enrolment.

1. Uncomplicated: patients with dengue with warning signs who never progressed, did not develop shock or require ICU (n = 49, median age = 28)
2. Moderate-severe: patients with dengue who were admitted to the emergency department with dengue shock syndrome but did not require ICU or organ support (n = 52, median age = 27.5)
3. Severe: patients with severe dengue admitted to ICU, for multiple or prolonged shock and/or organ support (n = 31, median age = 30)

Additional 56 paediatric patients (aged less than 16 years) with over 1345 h of raw PPG data were part of the secondary analysis. Here we looked at the subset of this cohort which contained a 30-min follow-up recording captured 14–21 days after the illness onset to serve as a baseline, post-dengue state for comparison. Both the adult and paediatric datasets are summarized in Table 3.

**Table 3:** Summary of both patient cohorts.

	Adult cohort	Paediatric cohort
n	132	56
Age, median [Q1,Q3]	28.0 [21.0, 35.0]	11.0 [9.0, 13.0]
Sex, n (%)		
M	75 (56.8)	24 (42.9)
F	57 (43.2)	32 (57.1)
Length of hospitalisation (days), median [Q1,Q3]	4.5 [4.0, 6.0]	4.0 [3.0, 5.0]
PPG recording duration (hours), median [Q1,Q3]	11.7 [5.3, 14.2]	24.2 [20.5, 27.8]

**Fig. 2:**
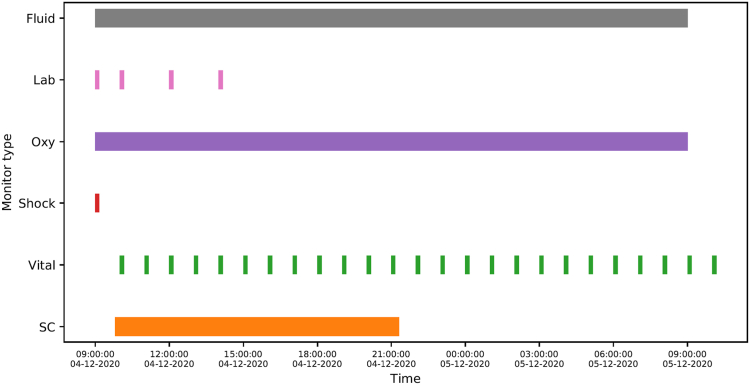
Visualisation of data from a patient suffering from severe dengue. The following clinical information is tracked: **Fluid** highlights the period of fluid resuscitation; **Lab** provides timestamps of blood tests; **Oxy** stands for the duration of supplemental oxygen therapy; **Shock** signifies the time clinical shock was determined by the attending physician; **Vital** are timestamps for vital sign collection (hourly) and **SC** is the duration of continuous PPG waveform measurement using a SmartCare wearable device.

Patients with moderate-severe and severe disease in ED/ICU were monitored more intensively as part of routine medical care. An illustration of such a clinical dataset for a severe patient, including the duration of captured raw waveforms can be found in Fig. 2. For uncomplicated patients, vital signs were taken at least every 6 h together with daily blood tests.

The continuous PPG data was processed and cleaned using the data quality pipeline, classifying waveform segments into one of the 4 categories: (1) excellent, (2) good, (3) poor and (4) completely unusable. Only the first 2 categories were useable for further analysis and patients with more than 10% of unusable segments were removed from analysis. The Supplemental Fig. S2a–d shows examples of each data quality class.

**Fig. 3:**
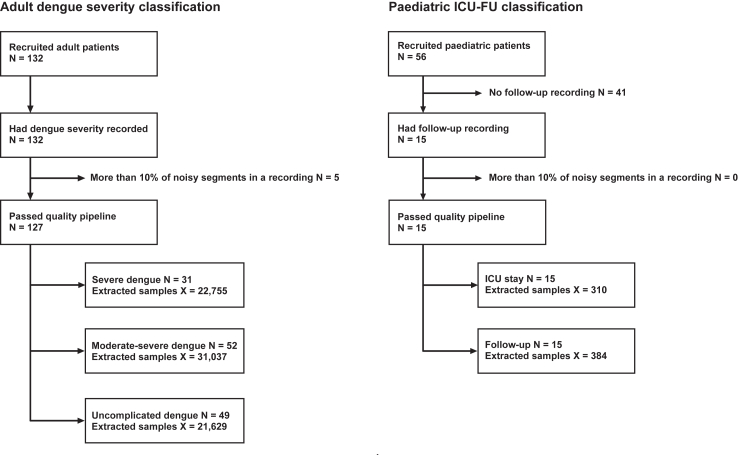
Participant inclusion flowcharts for both investigated research objectives. The N denotes number of participants, whereas X stands for number of acceptable quality 1-min segments extracted from the raw PPG data.

For the primary investigation, all available adult patient cohort data were applicable for the analysis. This cohort was split into 3 categories (uncomplicated, moderate-severe and severe) by the attending clinician, according to the disease progression during the enrolment period. These three categories were then used as classes for model development. After cleaning the dataset and assessing data quality, 127 patients were used for model development using 1223 h of continuous PPG data (Fig. 3a).

The secondary investigation evaluated the difference in PPG waveform features between acute disease (admission to ICU) and post-disease healthy state (follow-up). The dataset for this question only contained a subset of the whole paediatric cohort as not everyone had the follow-up recordings present. It was therefore treated as a binary classification problem, grouping all follow-up and ICU recordings into their respective classes. In total, data from 15 paediatric patients have been analysed, using all 7.27 h of available follow-up data and the same duration extracted from the ICU data for balanced class distribution (Fig. 3b).

### Classification between severity classes in adult patients with dengue

**Fig. 4:**
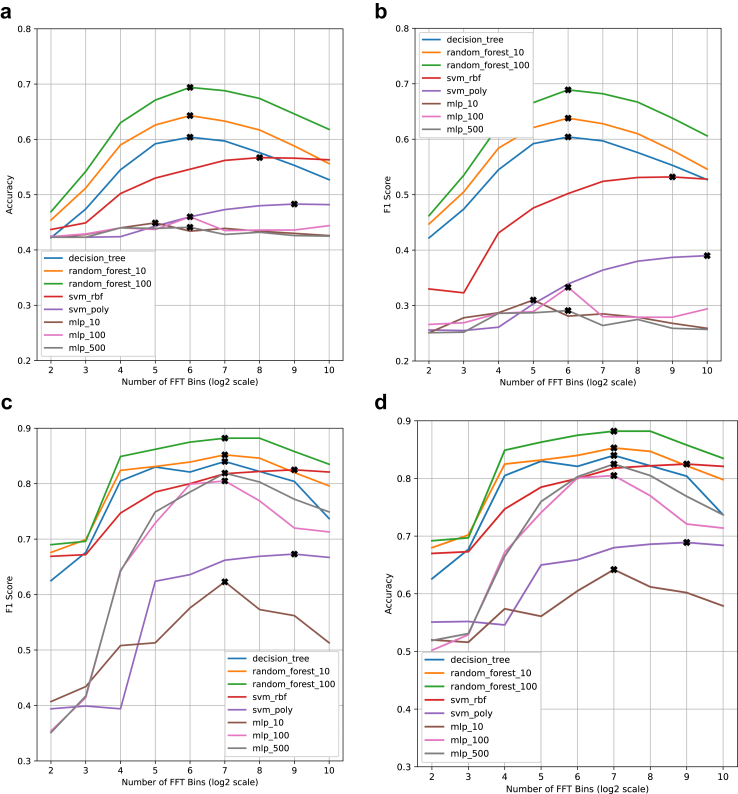
Baseline model performance charts across frequency bins, showing the clear peaks in performance. The plotted points correspond to the final metric after 5-fold cross-validation for the given model type and frequency resolution combination. (a) accuracy and (b) weighted F1 score for adult severity classification as a function of frequency resolution. (c) accuracy and (d) weighted F1 score for paediatric ICU-FU classification as a function of frequency resolution. Frequency resolution is quantified through the logarithm of the number of frequency bins.

When investigating the primary research objective, the best-performing baseline model was a random forest with 100 estimators and achieved an accuracy of 0.694, macro precision of 0.709 and weighted F1 score of 0.689. For the baseline, frequency domain feature set, we have also shown that there is an optimal frequency binning approach at 64 bins. This led to 7.6% and 8.3% improvements in accuracy and F1 score compared to 1024 frequency bins (Fig. 4a–b).

All baseline model performance results are summarized in Table 4. The achieved classification accuracy of 69.4% provides a solid initial performance considering the minimum computation complexity requirements for this type of model.

**Table 4:** Baseline model performance on time-domain and frequency-domain inputs for adult dengue severity classification.

Variation	FE domain	Model name	Accuracy	Macro precision	Macro recall	Macro F1	Weighted F1
10 wavelength Segment	Time-domain	DT	0.431	0.421	0.421	0.421	0.431
		RF (10)	0.486	0.479	0.454	0.455	0.472
		**RF (100)**	**0.548**	**0.586**	**0.503**	**0.504**	**0.523**
		SVM (RBF)	0.498	0.534	0.434	0.404	0.430
		SVM (poly)	0.440	0.501	0.359	0.269	0.435
		MLP (10)	0.478	0.479	0.430	0.419	0.444
		MLP (100)	0.442	0.430	0.428	0.428	0.440
		MLP (500)	0.461	0.449	0.444	0.445	0.457
5 wavelength Segment (truncated)	Time-domain	DT	0.434	0.426	0.426	0.426	0.435
		RF (10)	0.494	0.490	0.466	0.468	0.483
		**RF (100)**	**0.553**	**0.585**	**0.512**	**0.515**	**0.532**
		SVM (RBF)	0.502	0.548	0.445	0.424	0.452
		SVM (poly)	0.468	0.501	0.386	0.335	0.378
		MLP (10)	0.494	0.515	0.443	0.434	0.458
		MLP (100)	0.461	0.451	0.447	0.448	0.459
		MLP (500)	0.476	0.467	0.461	0.463	0.473
Best performing Model frequency Bins	Frequency-domain	RF (100)–4	0.469	0.454	0.446	0.446	0.462
		RF (100)–8	0.542	0.541	0.519	0.524	0.535
		RF (100)–16	0.630	0.636	0.606	0.613	0.624
		RF (100)–32	0.671	0.685	0.646	0.656	0.666
		**RF (100)–64**	**0.694**	**0.709**	**0.669**	**0.680**	**0.689**
		RF (100)–128	0.688	0.704	0.662	0.672	0.682
		RF (100)–256	0.674	0.692	0.646	0.646	0.667
		RF (100)–512	0.646	0.667	0.616	0.616	0.638
		RF (100)–1024	0.618	0.641	0.584	0.592	0.606

For deep learning CNN model implementation, we leveraged the large size of the adult cohort dataset and experimented with varying STFT window sizes. In addition to the sample window length of $66.\bar{6}$ seconds used for the baseline models, we have reduced the size to 1/8 of the original and increased it to 32 times the original length. The best-performing CNN classifier used 1/8 of the baseline STFT length and achieved an accuracy of 0.780, macro precision of 0.785 and weighted F1 score of 0.780, improving the overall classification accuracy by 8.6% compared to the best-performing baseline model. This suggests that a large number of samples has a higher effect on the overall performance compared to the STFT duration as the performance of other CNN variations was significantly lower (Table 5).

**Table 5:** Performance metrics for the best severity CNN model found by the optimiser for different sizes of the STFT.

FE domain	Model name	Accuracy	Macro precision	Macro recall	Macro F1	Weighted F1	# Training samples
STFT	CNN–1	0.689	0.709	0.665	0.676	0.684	9094
	**CNN–1/8**	**0.780**	**0.785**	**0.771**	**0.776**	**0.780**	**75,443**
	CNN -1 (32 windows)	0.615	0.629	0.591	0.599	0.609	226

Size is represented by the number by which the STFT window length is multiplied (e.g. 1/4 represents a quarter of the normal 10-wavelength-long window).

60 iterations of Bayesian optimization were performed to obtain optimal CNN parameters for each STFT configuration (Table 6). Here, we found that the best-performing CNN model contained a single dense layer with 4 nodes. Compared with the optimization results for other sizes of STFT windows, the lower dense layer stage complexity produced better classification performance.

**Table 6:** Model parameters for each of the models shown in Table 5.

	1	1/8	1 (32 windows)
Number of CLs	5	5	5
Kernel Size	(4, 4)	(4, 4)	(4, 4)
Number of DLs	3	1	2
Number of Nodes per DL	32	4	4
Learning Rate	5e − 3	6e − 4	7e − 4
Number of Filters of 1st CL	4	8	4

We have observed that the reduction in dataset size caused by prolonging the STFT window outweighed the potential gain in temporal variations and low-frequency features leading to degradation in the final performance of the model.

### Classification between paediatric patients with dengue in ICU and during follow-up after recovery

The secondary objective focused on a subset of paediatric patients whose available dataset contained a follow-up recording after discharge. To keep the binary class distribution balanced, we have used the shorter follow-up recordings to dictate the total number of samples used for model development. Despite the lower number of samples available, the best-performing frequency-domain baseline model was again a random forest with 100 estimators and achieved an accuracy of 0.882, macro precision of 0.881 and weighted F1 score of 0.882. Baseline model performance peaked between 128 and 256 frequency bins (Fig. 4c–d and Table 7).

**Table 7:** Baseline model performance on time-domain and frequency-domain inputs for ICU vs FU classification.

Variation	FE domain	Model name	Accuracy	Macro precision	Macro recall	Macro F1	Weighted F1
10 wavelength Segment	Time-domain	DT	0.614	0.611	0.609	0.609	0.613
		RF (10)	0.654	0.655	0.636	0.632	0.642
		**RF (100)**	**0.730**	**0.732**	**0.721**	**0.722**	**0.722**
		SVM (RBF)	0.675	0.673	0.663	0.664	0.670
		SVM (poly)	0.594	0.637	0.553	0.495	0.518
		MLP (10)	0.593	0.585	0.553	0.582	0.587
		MLP (100)	0.620	0.616	0.612	0.612	0.618
		MLP (500)	0.628	0.627	0.624	0.622	0.626
5 wavelength Segment (truncated)	Time-domain	DT	0.633	0.630	0.630	0.629	0.633
		RF (10)	0.677	0.681	0.664	0.662	0.669
		**RF (100)**	**0.753**	**0.754**	**0.746**	**0.747**	**0.751**
		SVM (RBF)	0.719	0.720	0.707	0.708	0.715
		SVM (poly)	0.650	0.677	0.618	0.601	0.617
		MLP (10)	0.642	0.638	0.636	0.635	0.640
		MLP (100)	0.666	0.664	0.661	0.661	0.665
		MLP (500)	0.665	0.662	0.661	0.660	0.664
Best performing Model frequency bins	Frequency-domain	RF (100)–4	0.692	0.689	0.685	0.685	0.690
		RF (100)–8	0.697	0.696	0.692	0.692	0.696
		RF (100)–16	0.849	0.848	0.847	0.847	0.849
		RF (100)–32	0.863	0.862	0.861	0.861	0.862
		RF (100)–64	0.875	0.875	0.874	0.874	0.875
		**RF (100)–128**	**0.882**	**0.881**	**0.881**	**0.880**	**0.882**
		**RF (100)–256**	**0.882**	**0.882**	**0.880**	**0.880**	**0.882**
		RF (100)–512	0.858	0.859	0.856	0.856	0.858
		RF (100)–1024	0.835	0.837	0.831	0.832	0.835

To combat the negative effects of a small number of training samples due to short follow-up recordings, we reduced the STFT window size in steps (Table 8) and retrained the model. Repeating the Bayesian optimization approach for each model iteration leads to the best performance again achieved at 1/8 of the original length. The achieved metrics of 0.892 and 0.891 for accuracy and macro precision respectively (Table 9) indicate excellent classification capabilities of the model and clear difference between the ill and healthy state in paediatric patients.

**Table 8:** Training set size, STFT shape for different sizes of the STFT input and CNN model parameters.

	1	1/2	1/4	1/8
# Training Samples	79	168	349	707
Number of Convolutional Layers	2	5	6	5
Kernel Size	(2, 2)	(3, 3)	(3, 3)	(3, 3)
Number of Dense Layers	2	2	1	1
Number of Nodes per Dense Layer	16	4	32	32
Learning Rate	2e − 3	8e − 4	2e − 3	8e − 4
Number of Filters of 1st Conv. Layer	4	8	8	8
STFT Shape	(3286, 9)	(1643, 9)	(822, 9)	(411, 9)

Size is represented by the number by which the full STFT length, as computed from a 10-wavelength long signal, is multiplied (e.g. 1/4 represents a quarter of the full STFT length).

**Table 9:** Performance metrics for the best ICU vs FU CNN model found by the optimiser for different sizes of the STFT.

FE domain	Model name	Accuracy	Macro precision	Macro recall	Macro F1	Weighted F1
STFT	CNN–1	0.760	0.776	0.744	0.744	0.751
	CNN–1/2	0.791	0.798	0.783	0.785	0.789
	CNN–1/4	0.827	0.833	0.823	0.824	0.826
	**CNN–1/8**	**0.892**	**0.891**	**0.891**	**0.891**	**0.892**

Size is represented by the number by which the STFT window length is multiplied (e.g. 1/4 represents a quarter of the normal 10-wavelength-long window).

### Baseline models interpretation

To understand which underlying features contributed the most to the classification result, we have conducted a permutation-based feature importance investigation.^21^ The analysis was only conducted for the best performing baseline models for each investigated objective. In both cases, these involved frequency domain features split into 64 or 128 bins as explained in the Methods section.

In the primary investigation of dengue severity, the best performing random forest used 64 frequency bins. From these, the 4 most impactful features occurred at 0, 1.27, 1.9 and 3.17 Hz. Translating these into clinical context, 0 Hz corresponds to the static bias of the PPG waveform, influenced by the absorptivity of the non-pulsatile parts of the tissue. With the sensor placed on the finger, the main contributing factor to this bias is the overall finger thickness. This can be both referenced to the overall BMI of the patient as well as to the severity of the disease as extremities can get swollen due to fluid therapy. Both 1.27 and 1.9 Hz corresponds to a regular heart-rate signal (76.2 and 114 bpm respectively), while the most influential feature at 3.17 Hz corresponds to a significantly elevated heart-rate of 190 bpm.

In the case of the ICU-FU investigation, the most influential feature is the static bias, which can point to the higher variability in finger thickness in children. The remaining features share importance around the regular and elevated heart-rate frequencies.

Based on these results, we can argue that the random forest models have learned to pick up on combinations between heart-rate and waveform bias to devise a set of complex rules for severity classification. With 100 estimators, the complexity of these models is beyond the capability of human decision makers. The complexity further raises in the case of the CNN approach, where similar interpretability analysis becomes obsolete due to changes in input feature representation. The full feature importance results for both severity and ICU-FU best-performing random forest models can be found in Supplemental Figure S3.

## Discussion

The ability of the models to reliably distinguish between various stages and illness severity in dengue is likely to be of clinical value in supporting individual patient assessment in hospitals. The investigated classification objectives aimed to tackle the issues with overcrowding during seasonal dengue epidemics. With the best-performing frequency domain algorithm only requiring 66 s of continuous data for classification, the model can be run repeatedly to provide a continuous assessment of the patient's clinical status during their hospital stay as well as at home. This is in contrast with existing clinical care in which patients in general wards may undergo vital signs monitoring every 6–8 h. The wearable sensor used in our study costs around 150 USD per unit and will likely be cost-effective for implementation in the LMIC healthcare setting. The scalable nature of wearables deployment, coupled with intrinsic connectivity allows for a means for patient monitoring which can cope with periods of high workload for healthcare staff, such as during large dengue outbreaks.^22^ By further optimizing the model to minimize the false negative rate of severe dengue classification, the developed model can be used to give the nursing team up-to-date information about the expected state of each patient, shifting focus to the most critical patients while allowing the uncomplicated ones to stay at home until their state worsens. This mass-deployable and low-cost wearable system can therefore provide a perceived “second set of eyes” for patients and clinicians, overcoming staffing issues and having the potential to greatly improve the standard of care.

The classification accuracy of 0.78 and 0.89 for the primary and secondary analyses respectively clearly showcase a relationship between dengue severity and wearable sensor PPG data, opening up avenues for scalable patient monitoring. Only marginal improvements in performance between STFT-CNN and frequency-domain random forest model for the paediatric analysis point to the sufficiency of the less complex model, allowing implementation on cost-effective hardware. It also shows that the added temporal variation introduced by the STFT does not provide enough information to improve classification performance significantly. The argument changes when looking at the adult cohort where the STFT-CNN approach improves the metrics by more than 8%. This confirms that larger datasets are a better fit for a deep-learning model.

Despite the promising results in dengue severity assessment, several substantial limitations warrant acknowledgement. One critical aspect revolves around the inherent “black-box” nature of the machine learning models, particularly regarding approaches from the deep learning paradigm, such as the Convolutional Neural Networks (CNNs) used. This opacity poses challenges in interpreting how the model arrives at its predictions, limiting the clinical utility of the algorithm. Based on the performance and interpretability of the baseline models, we can assume that the CNN network used similar set of critical frequencies to perform classification, with the performance improvements due to the spatial features of the constructed STFT frames. The generalizability of the results is further constrained due to the homogeneous ethnic composition of the cohort, primarily from Vietnam. On one hand, this constraint helps mitigate the effects of varying skin tones on PPG sensing while reducing application to the wider population. Employing PPG sensors in clinical settings poses its own set of limitations. Factors such as motion artefacts and environmental conditions can impact signal quality, potentially compromising the reliability and accuracy of data collected. Additional operational requirements on device provisioning and maintenance by the nursing staff need to be evaluated before any large-scale deployment in the affected regions. Furthermore, studies of this nature suffer from limitations in recruitment, including sample size constraints and specific inclusion criteria, that limit the diversity of the acquired dataset. It is important to note that some parts of the clinical dataset have not been used when formulating the research objectives. Namely, the duration and amount of fluid used during resuscitation as well as the day of illness when the PPG was taken. Both of these introduce an additional degree of inaccuracy as the acquired PPG signal would be influenced by the duration and volume of fluid received. Enhancing future models by including these parameters can further improve the performance of the classifiers.

The outcome of this work provides baseline actionable information to assist clinicians with the day-to-day triage of patients with dengue. Work is currently underway by our research group in the development of a bespoke multi-wavelength PPG wearable capable of continuous monitoring of the cardiovascular and haemodynamic status of patients with dengue.^23^ The developed platform expands on the SmartCare sensor operation, adding additional wavelengths and sensing sites to provide the capability for non-invasive haematocrit and pulse pressure sensing. Further work will include investigating the role of wearables in ambulatory clinical management, whereby patients can be managed safely as outpatients or at home, thus allowing for the effective allocation of staffing resources to provide care for the sickest patients. Presented results of the research objectives in this work, combined with the feature-rich PPG dataset available for future investigation, are opening the way for a commercial end-to-end system for patient management in low-income and middle-income countries.

## Contributors

Conceptualization: VM, SK, DKM, HQC, JD, BH, SY and PG; formal analysis: VM, SK, and JD; funding acquisition: AHH, LT, SY and PG; methodology: VM, SK, HQC, DKM, BH, SY and PG; supervision: AHH, LT, SY and PG; writing—original draft: VM, SK, HQC and JD; writing—review and editing: SK, DKM, BH, AHH, SY and PG; data collection: HQC, NTG, VNTH, MTVH, KPNQ, HNV, TQP, HTT, TPV and TLTH; data annotation: HNV, TQP, HTT, TPV and TLTH. SK, HQC and DKM verified the underlying data (validation). All authors have read and agreed to the published version of the manuscript.

## Data sharing statement

The anonymised datasets analysed during the current study are available through the Oxford data repository as well as on reasonable request to the author (syacoub@oucru.org), as long as this meets local ethics and research governance criteria. The code can be accessed at https://github.com/vasilismanginas/dengue-severity-classification.

## Declaration of interests

The authors declare that the research was conducted in the absence of any commercial or financial relationships that could be construed as a potential conflict of interest.
