# Supplementary material for: Towards a machine-learning assisted non-invasive classification of dengue severity using wearable PPG data: a prospective clinical study
Source: eBioMedicine. 2024 May 29;104:105164. doi: 10.1016/j.ebiom.2024.105164 (PMC11167237; doi:10.1016/j.ebiom.2024.105164)
Supplement: Supplementary Figures [file mmc1.docx]

**Towards a machine-learning assisted non-invasive classification of dengue severity using wearable PPG data: a prospective clinical study**

Stefan Karolcik^1a^, Vasileos Manginas^1a^, Ho Quang Chanh^c^ , John Daniels^a^ , Nguyen Thi Giang^c^ , Vu Ngo Thanh Huyen^c^ , Hoang Minh Tu Van^c^ , Khanh Phan Nguyen Quoc^c^ , Bernard Hernandez^a^ , Damien K Ming^b^ , Nguyen Van Hao^c^ , Phan Tu Qui^c^ , Huynh Trung Trieu^c^ , Luong Thi Hue Tai^c^ , Alison H Holmes^b^ , Louise Thwaites^c^ , Phan Vinh Tho^c^ , Sophie Yacoub^2c^ , Pantelis Georgiou^2a^ , on behalf of the Vietnam ICU Translational Applications Laboratory (VITAL) investigators

^a^Centre for Bio-Inspired Technology, Imperial College London, South Kensington campus, London, SW7 2AZ, United Kingdom

^b^Centre for Antimicrobial Optimisation, Imperial College London, Hammersmith campus, London, W12 0NN, United Kingdom

^c^Oxford University Clinical Research Unit (OUCRU), Hospital for Tropical Diseases, Ho Chi Minh City, 700000, Vietnam

**Supplemental Figures**


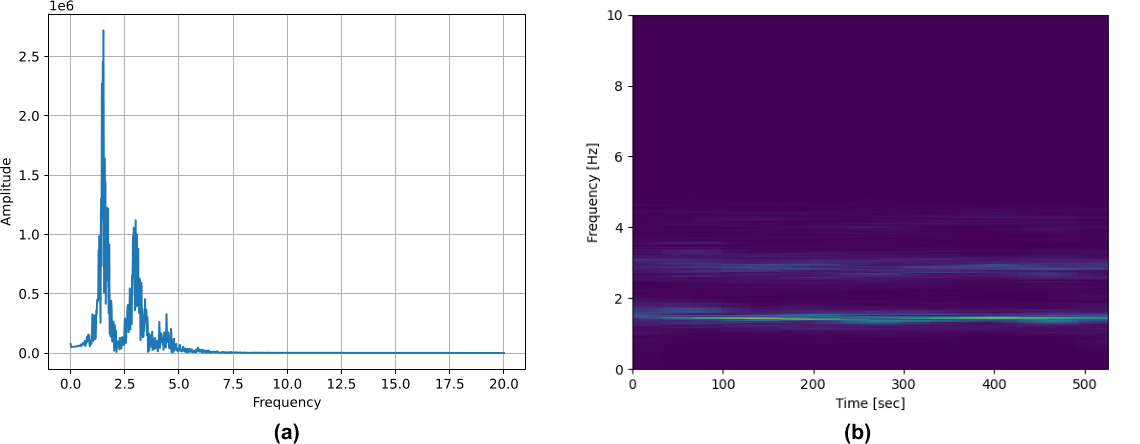


**Supplemental Figure 1:** PPG signal samples after feature extraction. (a) Magnitude of truncated FFT half-spectrum for the given PPG segment (b) Example STFT for the given PPG window constructed using 8 FFT windows spanning 525.6 seconds.


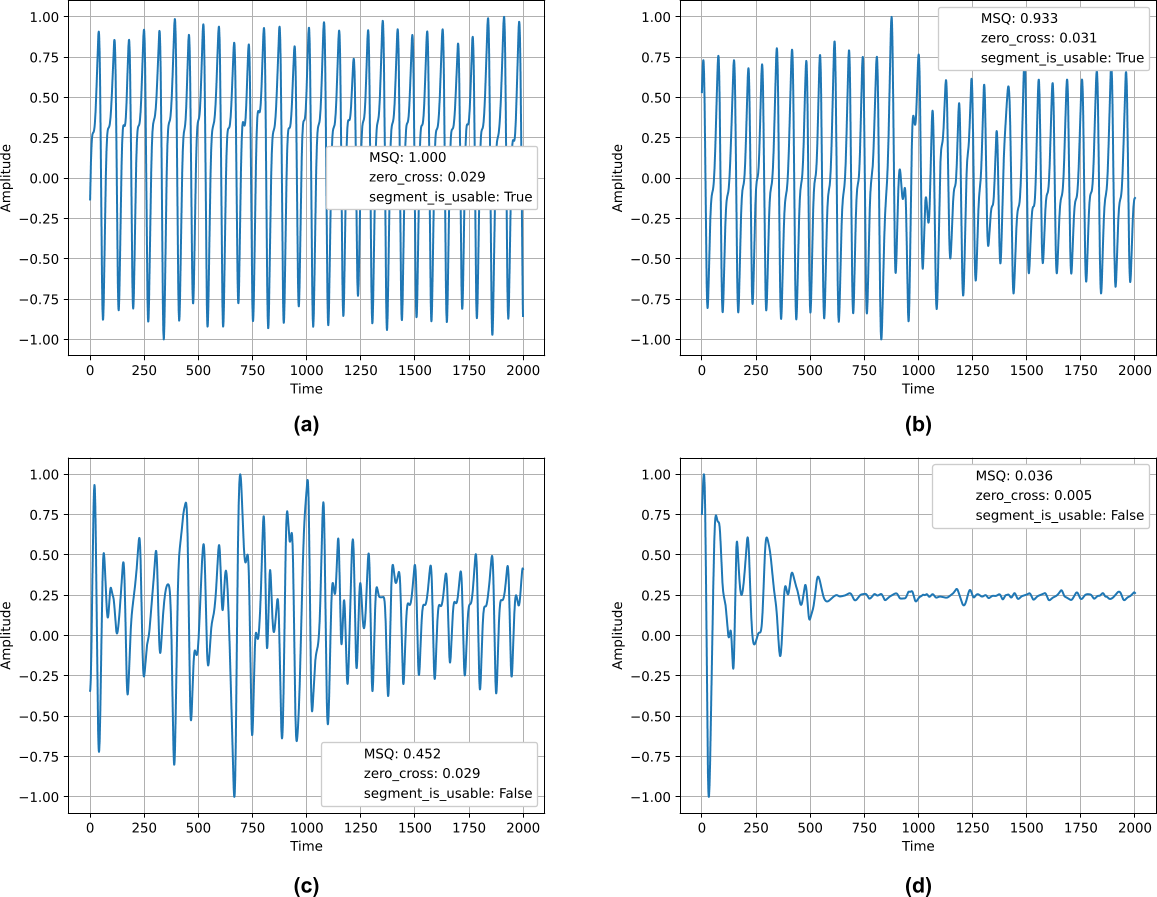


**Supplemental Figure 2:** Raw PPG signal quality evaluation using

$$Z_{SQI}$$

 and

$$M_{SQI}$$

 on a 20 second segment. Using SQI thresholding, the segments are classified into one of 4 classes: (a) excellent, (b) good, (c) poor, (d) completely unusable. Only good or excellent segments are used in further analysis.


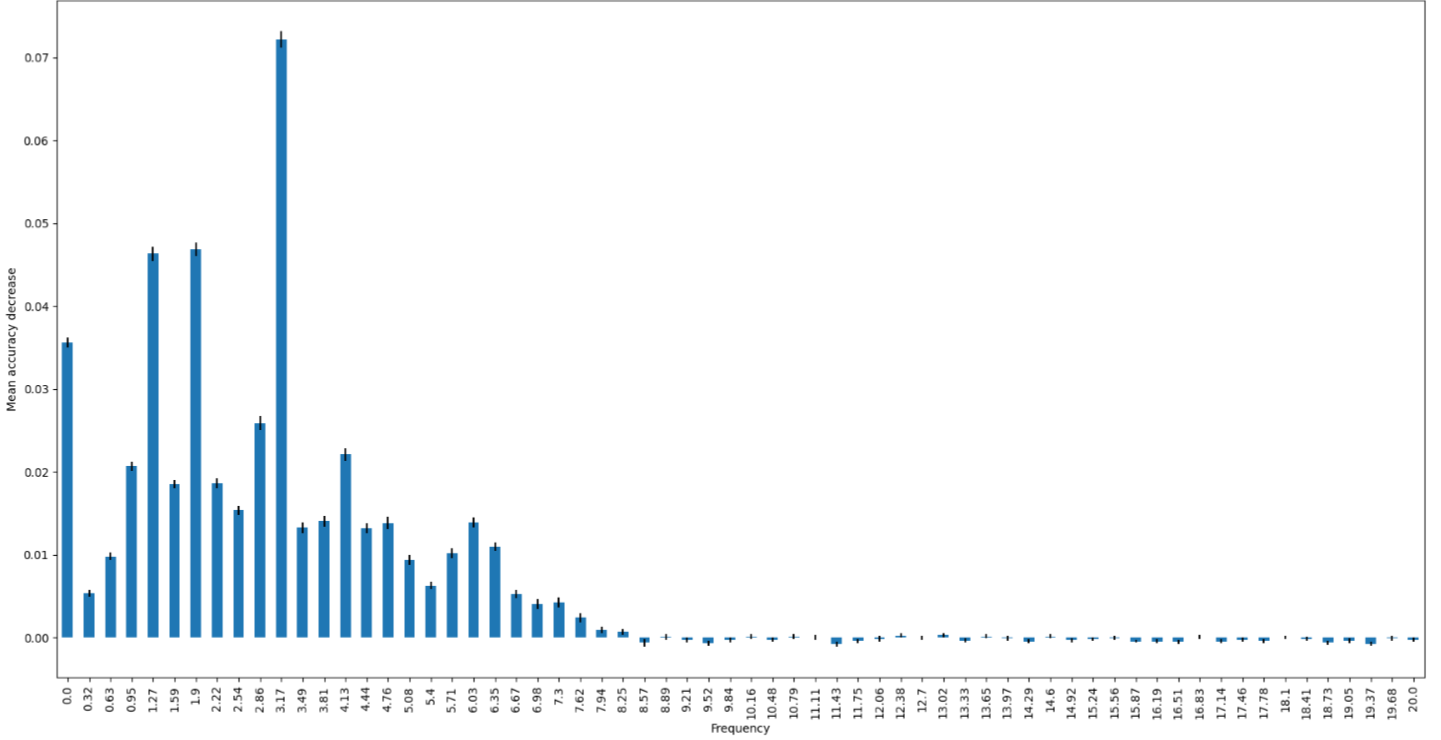


(a)


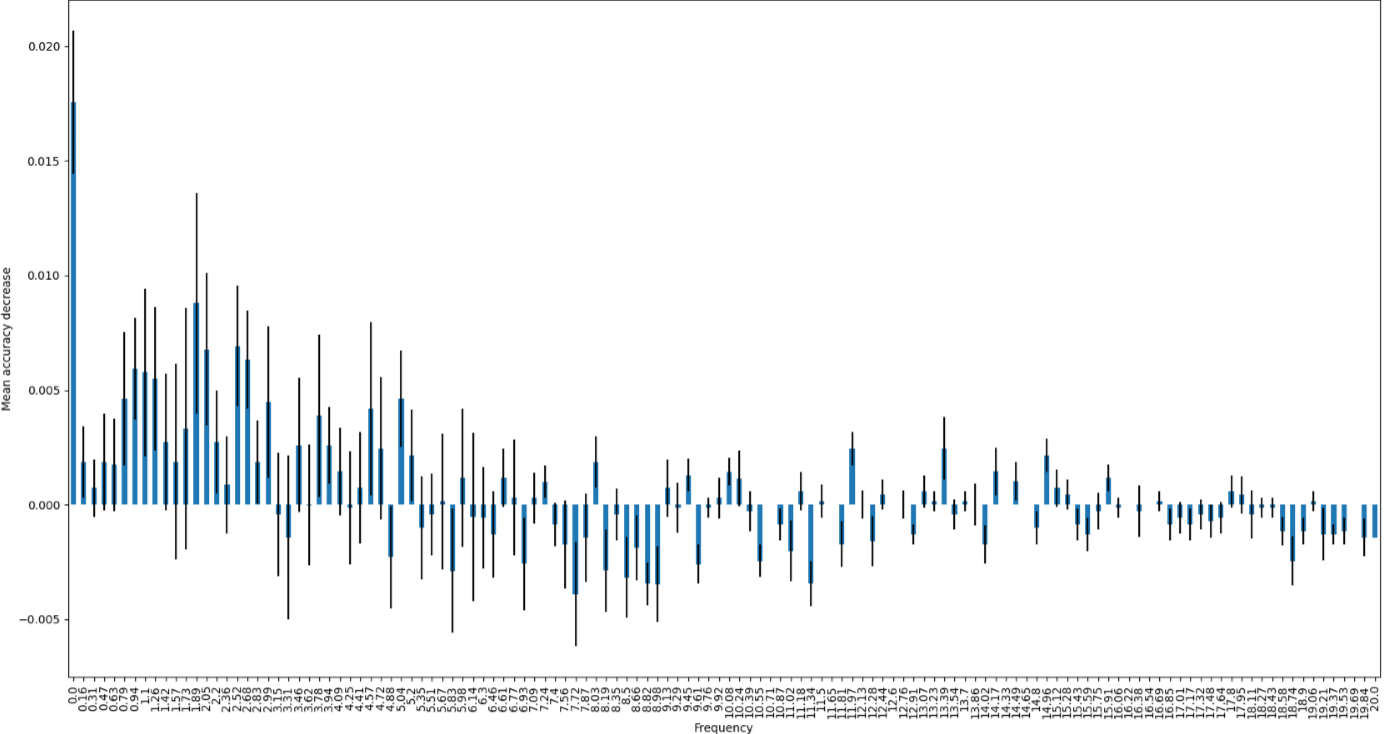
(b)

**Supplemental Figure 3:** Feature importance using feature permutation for the best performing baseline models. (a) severity classification with 64 frequency bins and (b) ICU-FU classification with 128 frequency bins. Each bar shows how much a given frequency bin weighed on the final classification result. The figures are averaged across the 5 folds of cross-validation.
