# Supplementary figures and images for: Towards a machine-learning assisted non-invasive classification of dengue severity using wearable PPG data: a prospective clinical study
Source: eBioMedicine. 2024 May 29;104:105164. doi: 10.1016/j.ebiom.2024.105164 (PMC11167237; doi:10.1016/j.ebiom.2024.105164)

**a**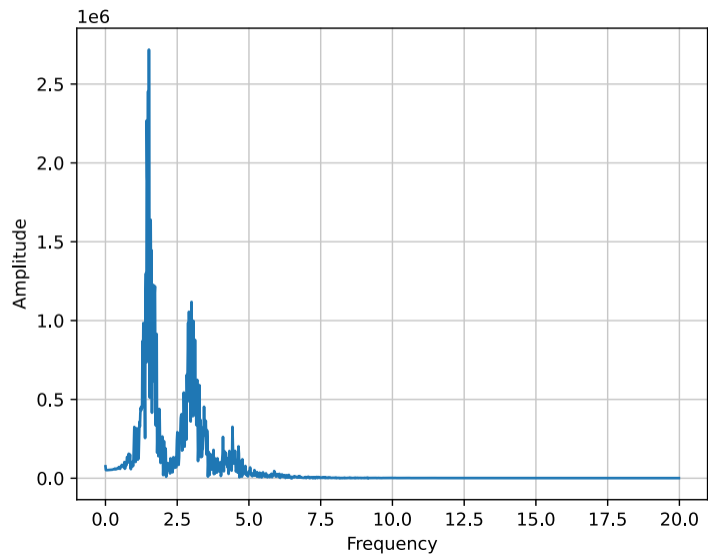**b**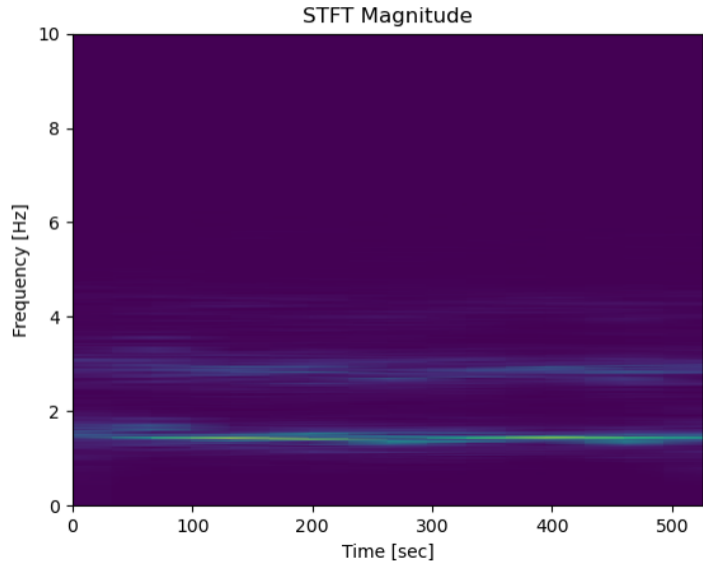

Supplement: Supplementary Figure S1 [file mmc2.pdf]

**a**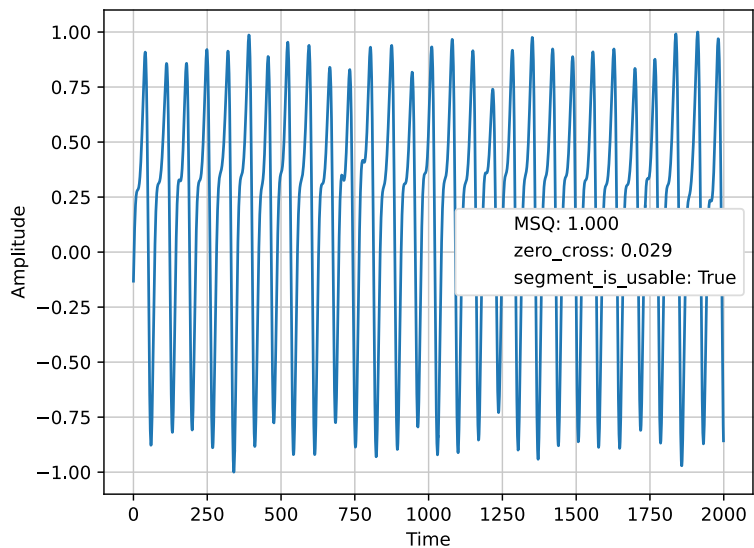**b**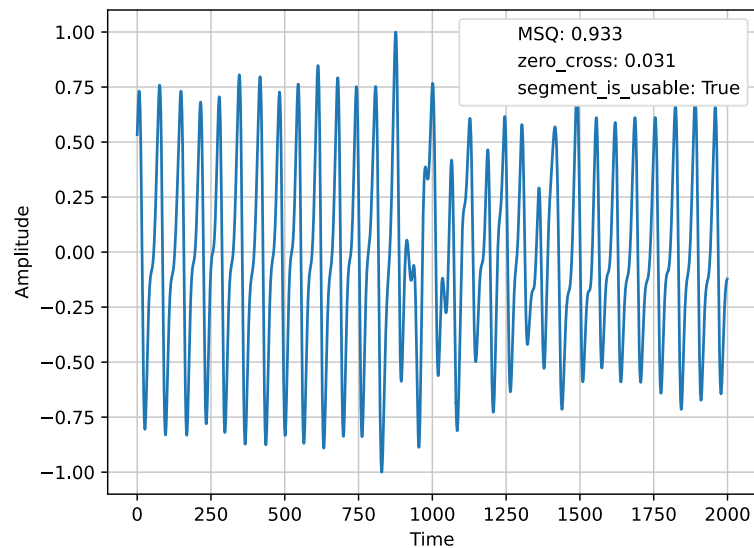**c**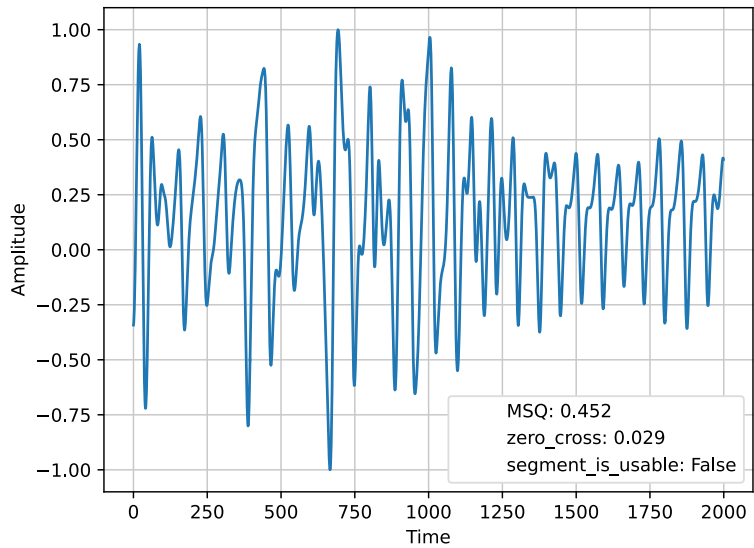**d**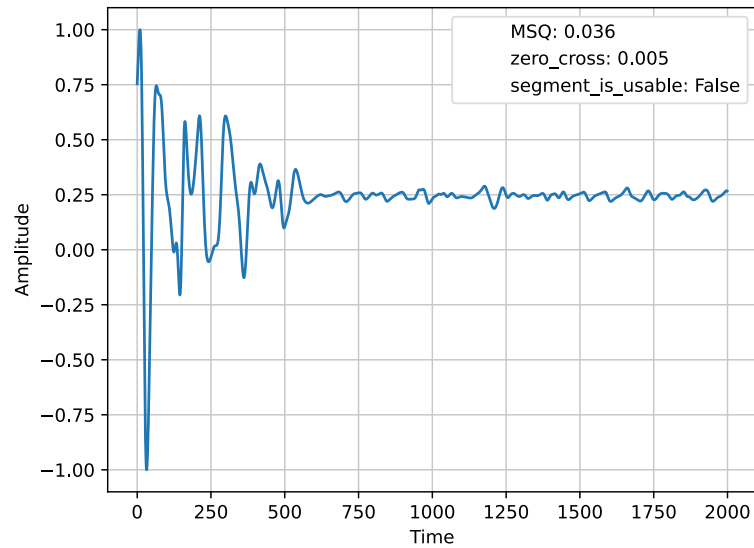

Supplement: Supplementary Figure S2 [file mmc3.pdf]
